# Exploring a Complex Interplay: Kidney–Gut Axis in Pediatric Chronic Kidney Disease

**DOI:** 10.3390/nu15163609

**Published:** 2023-08-17

**Authors:** Adriana Mocanu, Roxana Alexandra Bogos, Tudor Ilie Lazaruc, Laura Mihaela Trandafir, Vasile Valeriu Lupu, Ileana Ioniuc, Mirabela Alecsa, Anca Ivanov, Ancuta Lupu, Iuliana Magdalena Starcea

**Affiliations:** 1 Pediatrics Department, “Grigore T. Popa” University of Medicine and Pharmacy, 700115 Iasi, Romania; 2 Nephrology Division, St. Mary’s Emergency Children Hospital, 700309 Iasi, Romania

**Keywords:** microbiota, children, gut–kidney axis, chronic kidney disease

## Abstract

The human intestinal microbiota is a highly intricate structure with a crucial role in promoting health and preventing disease. It consists of diverse microbial communities that inhabit the gut and contribute to essential functions such as food digestion, nutrient synthesis, and immune system development. The composition and function of the gut microbiota are influenced by a variety of factors, including diet, host genetics, and environmental features. In pediatric patients, the gut microbiota is particularly dynamic and vulnerable to disruption from endogenous and exogenous factors. Recent research has focused on understanding the interaction between the gut and kidneys. In individuals with chronic kidney disease, there is often a significant disturbance in the gut microbiota. This imbalance can be attributed to factors like increased levels of harmful toxins from the gut entering the bloodstream, inflammation, and oxidative stress. This review looks at what is known about the link between a child’s gut–kidney axis, how dysbiosis, or an imbalance in the microbiome, affects chronic kidney disease, and what treatments, both pharmaceutical and non-pharmaceutical, are available for this condition.

## 1. Introduction

The human gut microbiota is a complex ecosystem that is essential for maintaining health and preventing disease. Hippocrates, in 400 BC, distinctly stated, “All diseases begin in the gut” [1]. The gut microbiota comprises approximately 10–100 trillion microorganisms [2,3] residing in the human intestine, forming a symbiotic relationship [4,5]. These microorganisms include bacteria, viruses, fungi, archaea, and unicellular eukaryotes, collectively possessing 3.3 million genes [6]. The gut microbiota can also be characterized based on its functional diversity, which relates to its impact on systemic immunity and host defense against intestinal pathogens [1,6]. The metabolism of microorganisms includes proteins, lipids, carbohydrate fermentation, bile acids, and vitamin synthesis [7,8]. Various factors, such as age [9], medications, allergens, sanitation, and different contagious illnesses [10], influence the variety, homogeneity, and enrichment of the microbiota [11,12].

The microbial communities that colonize the human gut are extremely diverse and highly personal [13]. These microorganisms play a vital role in the digestion of food, the synthesis of vitamins and other nutrients, and the development and function of the immune system. Gut microbiota structure and role are influenced by a variety of agents, including dietary habits, host genetics, and factors related to environment, with recent research exploring the ecological aspects that shape these microbial communities.

Although the mature adult gut microbiota is considered to be relatively stable, the developing infant gut microbiota (IGM) is constantly being reshaped, being prone to perturbation by external factors [13]. One of the most significant factors that can disrupt the development of IGM is the use of antibiotics, which are typically prescribed at a higher rate during the first years of life. Therefore, their impact on the infant’s gut microbial architecture and host disease is becoming a key priority of current research [14]. Antibiotics can disturb the microbial equilibrium, and create conditions that favor the growth of harmful bacteria. This can increase susceptibility to infections and other conditions such as allergies. Besides their direct effects on the gut microbiota, antibiotics also contribute to development of antibiotic-resistant bacteria [15,16,17]. Gut microbes are recognized as a significant epidemiological source of resistance genes (resistome), previous research suggesting that the actual structure of gut-associated resistomes is still largely unknown and more diverse than previously thought [18,19]. 

Antibiotic resistance is a public health problem today that threatens to undermine the effectiveness of antibiotics in treating infectious diseases. When antibiotics are used excessively, they can kill sensitive bacteria, leaving behind a population of resistant bacteria that can multiply and spread. This phenomenon has the potential to give rise to the emergence of antibiotic-resistant strains.

Antibiotic prescription in infancy and childhood can lead to antibiotic resistance genes, posing a threat to effective disease treatment [20]. Preventive public health programs are crucial, and alternative strategies like probiotics, prebiotics, and dietary interventions are needed to preserve intestinal flora and reduce antibiotic use in agriculture [21].

Understanding the role of the early disruption of the human microbiome and its impact on disease development is an active area of research. Scientists are investigating interventions such as probiotics, prebiotics, and microbial therapies to restore or promote a healthy microbiome in infants. By optimizing the early establishment of the microbiome, it may be possible to mitigate the risk of certain diseases and support overall health and well-being from an early age.

Overall, the study of intestinal microbes and their interactions with antibiotics and other environmental factors is a rapidly evolving field with significant implications for human health and disease. By better understanding these complex interactions, we can develop more effective strategies for preserving the health and well-being of individuals and populations [22]. 

This literature survey is designed to assess the present state of knowledge the gut–kidney axis, the impact of the microbiome and dysbiosis on chronic kidney disease (CKD), and the available pharmacological and non-pharmacological treatment options for this condition.

## 2. CKD and Gut Microbiota

Up to 10% of the population worldwide is affected by CKD [23]. In recent years, researchers have focused on the gut–kidney interaction in CKD. Patients often experience a prevalent disturbance in their gut microbiota. This imbalance is connected to various factors, including elevated levels of gut-derived uremic toxins in the bloodstream, inflammation, and oxidative stress. These factors are closely associated with cardiovascular disease and an increased risk of morbidity and mortality. Targeting the gut microbiota through therapies could potentially prevent CKD and its associated health conditions [24,25]. Given that CKD can start in early life, even during the prenatal period in some cases such as severe obstructive malformations, Prune Belly syndrome, and polycystic diseases, it is crucial to initiate prevention and treatment as early as possible. Recent research suggests that the early disruption of the microbiota is considered significant in the onset and advancement of various diseases, as it affects crucial metabolic and immunomodulatory processes. Already established is the fact that there are differences in the quantity and quality of intestinal microflora in newborns depending on the method of delivery. [26,27,28,29]. Dysbiosis, which refers to abnormal alterations in the gut microbiota, can compromise the integrity of the intestinal barrier. This, in turn, can cause translocation of bacteria with the buildup of dysbiotic gut-derived metabolites, including urea, indoxyl sulfate (IS), and p-cresyl sulfate (PCS). These metabolic processes can result from inflammation mediated by specific immune cells, stimulating antibodies, immune complexes, and inflammatory factors overproduction. This immune response can cause inflammation and the infiltration of inflammatory cells, which can damage the renal parenchyma either directly or indirectly [30,31,32]. Treatment options include prebiotics, probiotics, postbiotics, and symbiotics, as well as interventions on diet and lifestyle [33].

### 2.1. Developmental Origins of Health and Disease and CKD

Developmental Origins of Health and Disease (DOHaD) is a concept proposing that early life experiences, including fetal development, can have long-term effects on an individual’s health later in life [34]. This field of research suggests that environmental factors and experiences during critical periods of development can influence the programming of physiological systems and increase susceptibility to certain diseases in adulthood. The DOHaD framework suggests that unfavorable circumstances in prenatal development, such as poor maternal nutrition, distress, exposure to toxins, or impaired placental function, can lead to alterations in fetal growth and development. The adaptations of the fetus to these adverse conditions during pregnancy may increase the risk of installing chronic diseases [35,36].

The number of nephrons a person has is determined during fetal development and remains relatively stable throughout life. Research suggests that individuals born with a lower number of nephrons, a condition known as a low nephron endowment, are more susceptible to developing kidney disease and consecutive hypertension later in life. The low nephron endowment theory proposes that individuals with a reduced nephron number have a limited capacity to compensate for age-related nephron loss or adapt to other insults such as high blood pressure or diabetes. For this reason, they may be more prone to developing kidney disease, including CKD, and hypertension, which is a leading cause of CKD [37].

Maternal protein restriction [38,39,40] and iron and vitamin A deficiency [41,42] have been identified as factors that can disrupt normal fetal nephrogenesis—the process of kidney development. Furthermore, a recent study revealed the significance of fetal programming in nephrogenesis by demonstrating the effects of maternal fasting for 16 h per day [43].

Understanding the relationship between low nephron endowment and increased risk of kidney disease and high blood pressure highlights the importance of early life interventions and preventive strategies. Identifying individuals with a low nephron endowment and implementing measures to preserve kidney health and manage blood pressure can be crucial in minimizing the burden of kidney disease and hypertension later in life.

### 2.2. Nitric Oxide (NO) Prenatal Deficiency and CKD

NO modulates a number of important physiological functions in the digestive system and appears to be a crucial mediator of tissue damage in a number of diseases. Patients with chronic kidney disease have lower levels of the antioxidant enzymes catalase and Cu-Zn superoxide dismutase [44]. This shows that inflammation and disruption of the epithelial tight junction by uremic toxins is linked to a weaker antioxidative system. Simultaneously, plasma concentrations of nitric oxide synthase, monocyte chemotactic protein 1, and COX-2 are elevated. Nitric oxide is known to significantly modulate a number of physiological processes. The mechanisms, through which NO plays various roles in organisms, include the modulation of sodium transporters, epigenetic regulation, and the influence of gut microbiota.

**Figure 1:**
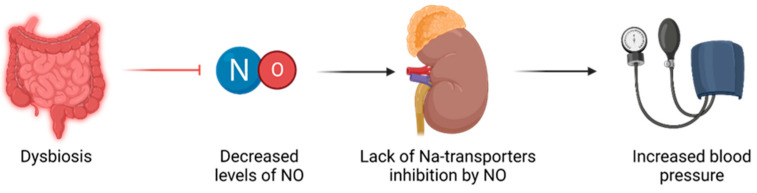
Relationship between dysbiosis and hypertension, mediated through NO imbalances.

- Sodium transporters: renal disease and high blood pressure have been linked to increased expression and activity of sodium transporters, leading to higher sodium reabsorption [44,45]. NO has been shown to inhibit the work of certain sodium transporters [46]. Therefore, it is thought that a deficient NO may fail to balance the impaired sodium transporters in the context of early life insults, ultimately contributing to programmed high blood pressure, as illustrated in Figure 1.
- Epigenetic regulation: Epigenetic mechanisms, such as histone alterations, DNA methylation, and RNAs of a non-coding nature play a role in developmental programming [47]. These mechanisms can influence gene expression patterns and contribute to long-term health outcomes. It is possible that NO signaling may impact epigenetic regulation, thereby influencing programming of hypertension and renal disease.
- Gut microbiota: The diversity of the gut microbiota is influenced by various factors, including genetics, comorbidities, and environmental factors like physical exercise, smoking, and medication use. However, it is undeniable that diet, dietary patterns, and specific components of the diet play a significant role in shaping the composition of the gut microbiota. These components refer to microorganisms that are not broken down, but can instead colonize the colon [48]. Moreover, the composition of the diet and the presence or absence of specific nutrients are crucial factors determining the rate at which these bacteria generate and the metabolic effects of the metabolites they produce [49].

Emerging evidence suggests a connection between hypertension development and dysbiosis of the gut microbiota [50,51]. Notably, insufficient NO has been proposed as a potential link between dysbiosis and hypertension [51]. 

Several recent investigations have elucidated potential interconnections between gut dysbiosis and the impairment of the nitric oxide pathway, as well as the dysregulation of the renin–angiotensin system, in relation to secondary hypertension [44,52]. The accumulation of uremic toxins in chronic kidney disease is a significant factor contributing to the increased risk of cardiovascular complications [53,54]. Besides asymmetric dimethylarginine (ADMA) and symmetric dimethylarginine (SDMA), endogenous inhibitors of nitric oxide synthase are significant uremic toxins that have a role in the development of cardiovascular disease during chronic kidney disease. SDMA is related to arterial hypertension in children and adolescents with CKD [55,56].

### 2.3. The Kidney–Gut Axis in CKD

**Figure 2:**
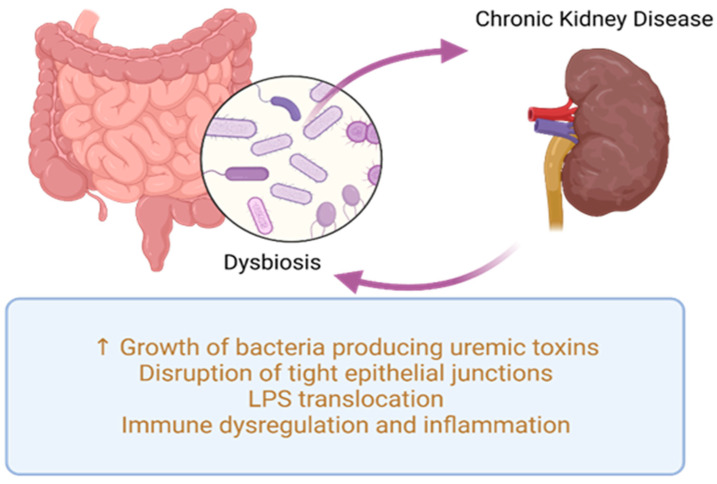
Common pathogenic processes in dysbiosis and CKD. LPS—lipopolysaccharides.

The relationship between gut microbiota and CKD is bidirectional and referred to as the kidney–gut axis [7]. This relationship is reciprocal: CKD can influence intestinal microbiome composition and potentially lead to dysbiosis, while dysbiosis in CKD patients can increase levels of uremic toxins, further exacerbating CKD progression, as shown in Figure 2. Recognizing the intestine as a potential factor in CKD-related complications, therapeutic approaches targeting gut microbiota will have a significant impact on CKD management.

**Figure 3:**
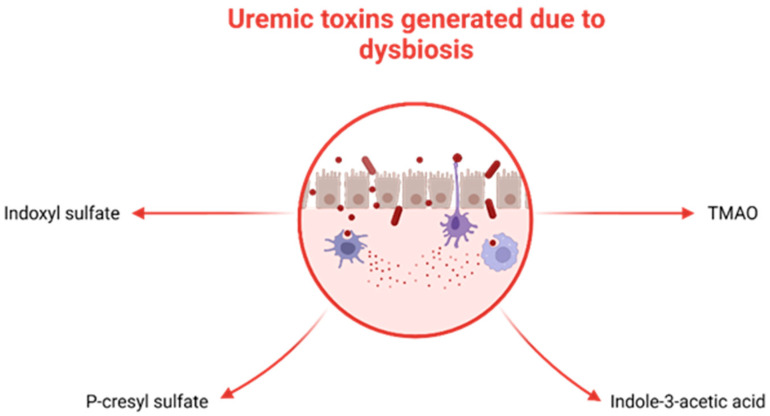
Uremic toxins generated by selected bacteria in the context of dysbiosis. TMAO.

Recent studies focused on adults with CKD have presented various mechanisms that establish a link between gut microbiota dysbiosis and kidney disease. These mechanisms include inflammation, impaired gut barrier function, changes in microbiota composition, immune response, accumulation of trimethylamine N-oxide (TMAO), disruptions in short-chain fatty acids (SCFA) and their receptors, as well as uremic toxins [23,57]. Dysbiosis fosters the proliferation of uremic toxin-generating bacteria (illustrated in Figure 3), such as p-cresyl sulfate (p-CS), indole-3-acetic acid (IAA), IS, and TMAO, which accumulate in individuals with CKD [58]. Additionally, dysbiosis disrupts the integrity of tight junctions in the epithelium, resulting in bacterial LPS displacement, impaired immune function, and the onset of inflammation [59,60].

CKD affects the integrity of the intestinal barrier by compromising tight epithelial junctions [61], primarily due to the presence of uremic toxins [23]. Consequently, there is an escalation in intestinal permeability, facilitating the displacement of LPS and pathogens through the digestive barrier. In individuals with CKD, intestinal flora stimulates the immune system by triggering the T-helper, which will amplify cytokine generation. Meanwhile, LPS can activate the innate immune response using the nuclear factor kappa B (NF-κB) and Toll-like receptor 4 (TLR4) pathways, leading to an inflammatory process and an immune response [23,57].

The presence of a leaky gut can lead to inflammation, malnutrition, and a faster progression of CKD [62,63]. Uremia has an important influence on the biochemical environment, favoring disruptions in gut microbiota and the intestinal barrier [30,31,64,65]. Moreover, the following also play a role in the development of dysbiosis: increased uric acid, inappropriate fiber consumption (decreased amounts of fruits and vegetables which can lead to hyperkalemia), and medication regimens (including antibiotics) [59].

In patients with CKD, the generation of uremic toxins has a negative impact on the growth of intestinal microbes. Studies have shown that individuals with end-stage kidney disease (ESKD) have lower diversity in their gut microbiota compared to healthy individuals [66]. Previous research has also suggested that CKD recipients tend to have decreased levels of good bacteria such as *Bifidobacterium* and *Lactobacillus* species [58].

Furthermore, there are various elements associated with CKD that contribute to an unbalanced gut microbiota. These factors encompass insufficient consumption of dietary fiber, malnutrition, metabolic acidosis, the utilization of antibiotics or other pharmaceuticals, augmented elimination of urea in the intestines, the buildup of uremic toxins, and reduced intestinal motility. These modifications in the uremic environment are linked to significant ramifications, including the advancement of CKD to end-stage renal disease, complications such as protein–energy wasting, and cardiovascular ailments, ultimately culminating in heightened mortality rates [67].

However, data are scarce regarding the impact of the kidney–gut axis in renal diseases of the minor population and the impact of intestinal dysbiosis on the modulation of pathological processes [23]. 

### 2.4. The Kidney–Gut Axis in Urinary Tract Infections

**Figure 4:**
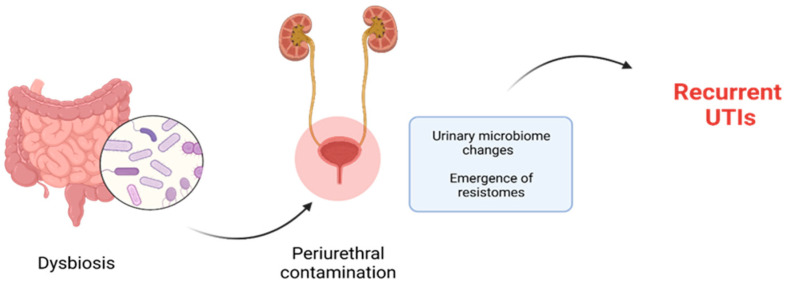
Dysbiosis promotes periurethral contamination with selected microorganisms involved in development of recurrent UTIs.

Periurethral contamination with specific uropathogenic bacteria, which are normally resident in the intestine, is recognized as playing a critical part in the pathology of urinary tract infection (UTI). This contamination is followed by the urethral colonization and the consequent ascension of the causative agent into the urinary bladder (Figure 4). Once in the bladder, the bacteria adhere to the uroepithelial cells using species-specific mechanisms, and then begin to multiply. Therefore, investigating the relationship between gut microbiota, bacteriuria, and UTI is an important area of research [68,69].

The previously well-established diagnostic and therapeutic approach for UTI has become less prominent with the discovery of a complex symbiotic microbiome in the healthy urogenital tract. Specifically, current evidence suggests that vaginal dysbiosis may lead to colonization by *Escherichia coli* and recurrent UTIs. Moreover, disruptions in the microbial flora of the urinary tract favor the onset of UTIs and other diseases of the urinary system, such as urinary lithiasis [70].

The recognition of the urinary microbiome’s role in urinary system health has sparked numerous research studies aiming to identify various classifications, including classes, orders, families, genera, and specific species. In the past decade, research involving the microbiome has sought to establish correlations between dysbiosis (microbial imbalance) in the gut bacterial community and a wide range of medical conditions, including gastrointestinal, respiratory, cardiac, neurological, psychiatric (and autism), metabolic, and cancer diseases [71]. One example of a disorder associated with gut–brain interactions is irritable bowel syndrome, which has shown an increasing incidence in recent years [72,73]. Furthermore, the human microbiome plays a role in impaired nutrient absorption, as seen in conditions such as celiac disease. This disorder is characterized by a microbial imbalance, with increased amounts of certain genera of Gram-negative bacteria, like *Bacteroides*, *Escherichia*, and *Prevotella*, and decreasing concentrations of good bacteria, such as *lactobacilli* and *bifidobacteria*. Moreover, individuals with celiac disease also exhibit dysbiosis involving viruses and fungi [74].

Although the gut microbiota of adults is considered to be relatively stable, the gut microbiota of developing infants and children is highly dynamic and susceptible to disturbances caused by external factors, such as exposure to antibiotics. It is well known that antibiotic therapy is one of the most commonly prescribed treatments in neonatal and pediatric populations.

The disruption caused by antibiotic therapy during the crucial development of children’s intestinal microbiota has significant implications for their health, leading to metabolic and immune disruptions [14]. Equally concerning is the possibility of enriching the reservoir of antibiotic resistance genes (“resistomes”), which can be transferred to pathogens [18,19], posing challenges in the treatment of infections, especially in vulnerable populations. This fact holds particular importance in the therapy of recurrent urinary tract infections.

Antibiotics used to treat UTIs can affect the intestinal microbiota, leading to a reduction in abundance and diversity, known as dysbiosis [75]. Similarly, recurrent exposure of the urinary microbiome to antibiotics could potentially result in alterations in the urinary microbial community, contributing to the emergence of resistomes [76]. Dysbiosis of the urinary microbiome is one of the main causes of recurrent UTIs. The increasing bacterial resistance to antibiotics encourages the search for non-antibiotic treatment options, including microbiome manipulation. Remodeling the urinary microbiome may help in controlling recurrent urinary tract infections and can serve as an important alternative to long-term antibiotic therapy [77]. Therefore, in pediatric patients with recurrent UTIs should be chosen a personalized management approach, specific to their clinical situation. 

Phytotherapy, recognized as a modern and effective approach for treating antibiotic-resistant bacterial infections, relies on a wide range of products such as natural compounds, vitamins, minerals, and probiotics. Two major mechanisms are involved in the antimicrobial properties of phytochemical compounds. Some directly induce bacterial cell death by altering the bacterial wall or inhibiting bacterial replication, while others interfere with microbial adhesion to urothelial cells [78]. 

Cranberry (*Vaccinium macrocarpon*) is a distinct source of polyphenols (flavonoids and phenolic acids). Polyphenols function during the bacterial adhesion phase to the urothelium by deactivating or inhibiting the adhesion of uropathogenic *E. coli* species, preventing bacterial colonization and the progression of UTIs [79,80]. 

### 2.5. The Kidney–Gut Axis in Urinary Lithiasis

Although urinary lithiasis is uncommon in children, the connection between intestinal flora and renal lithiasis has been described [81]. A meta-analysis revealed that the intestinal flora in patients with stone formation is marked by lower levels of *Prevotella*, *Prevotellaceae*, and *Roseburia*, and increased levels of *Enterobacteriaceae* and *Streptococcaceae* [81]. Urease-producing bacteria, such as *Proteus mirabilis*, *Klebsiella pneumoniae*, *Staphylococcus aureus*, *Pseudomonas aeruginosa*, *Providentia stuartii*, *Serratia*, and *Morganella morganii*, are always associated with the formation and recurrence of struvite stones [82].

Since urinary levels of oxalate, calcium, and uric acid are significant factors in the formation of kidney stones, an increased overall absorption in the gastrointestinal tract resulting from bacterial decomposition of these constituents of calculi could potentially influence their elimination through urine. Kidney stones composed of calcium oxalate are the most prevalent type. The absence of microbes capable of breaking down oxalate, such as *Oxalobacter formigenes*, has been associated with the formation of these stones [83]. The microbiota may also play an important role in the development of uric acid lithiasis, as under normal conditions one third of it is eliminated through the gut. Various data have demonstrated differences in the profile of intestinal flora between individuals with kidney stones and those without, further suggesting the crucial involvement of the intestinal microbiota in the formation of stones [81].

### 2.6. The Kidney–Gut Axis in Kidney Transplantation

Renal transplantation stands as the most effective treatment option for individuals diagnosed with end-stage renal disease. Despite the enhanced quality of life experienced by renal transplant recipients (RTRs) compared to individuals undergoing dialysis, they may encounter various challenges in the years following transplantation [84]. Recent studies have revealed that one in five RTRs experiences chronic diarrhea, which is associated with reduced quality of life, increased morbidity and mortality, and intestinal dysbiosis [85,86,87]. 

The use of immunosuppressive medications and frequent reliance on antibiotic therapy not only impacts the intestinal microbiome but also affects the urobiome [88]. These factors contribute to excessive growth of Escherichia coli strains and increased colonization by opportunistic pathogens [89].

### 2.7. The Kidney–Gut Axis in Other Kidney Diseases

Renal tubular impairment (acute tubular necrosis, tubulointerstitial nephritis, or idiopathic interstitial nephritis) is frequently observed in patients with inflammatory bowel disease, and the morbidity associated with renal manifestations in these cases can be as high as 20% [90]. Furthermore, deficiencies in intestinal immune tolerance lead to antigen absorption and activation of mucosa-associated lymphoid tissue, resulting in excessive deposition of abnormal IgA1 in the glomerular region, ultimately leading to IgA nephropathy [91]. The gut microbiota, through the B-cell activating factor of the TNF family, plays a key role in the development of IgA nephropathy [92].

## 3. Microbiota Modulatory Therapies in CKD Patients

**Figure 5:**
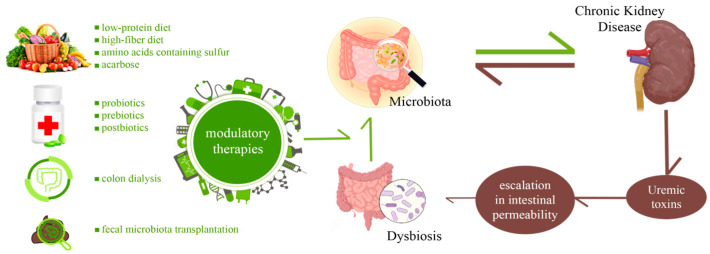
Microbiota modulatory therapies in CKD patients.

Promising strategies for preventing kidney disease involve potential therapies aimed at modulating the gut microbiota and microbial metabolites. In clinical practice, various gut microbiota-targeted therapies have been implemented for kidney disease, such as dietary interventions, probiotics, prebiotics, postbiotics, fecal microbiota transplantation (FMT), and phytotherapy [93], like we have exemplified in Figure 5.

### 3.1. Diet Intervention in Microbiota Modulation in Pediatric CKD

Dietary interventions serve as the first-line treatment for CKD. A low-protein diet has the ability to modify the gut microbiome and regulate the production of uremic toxins, potentially slowing down the progression of CKD and related cardiovascular diseases [52,94].

Consuming a high-fiber diet, which provides the necessary substrates for a healthy microbiota, may help prevent CKD progression by promoting the growth of bacteria that produce short-chain fatty acids [95]. The limited bioavailability of potassium and phosphorus in plant-based foods has led to recent changes in international dietary guidelines for children with chronic kidney disease (CKD). Fruits and vegetables high in potassium should no longer be inherently excluded from the diet of patients with advanced CKD, according to the new guidelines. However, further research is required to determine the health benefits of plant-based diets compared to omnivorous diets in children with CKD [96,97].

Studies have indicated that the consumption of amino acids containing sulfur can potentially benefit the advancement of CKD and its related complications [52]. However, there is limited available research on the link between gluten-free and dairy-free diets on the intestinal microbiome and the balance between Treg and TH17 cells in pediatric patients with steroid-resistant nephrotic syndrome. Only one study has investigated this specific topic [98]. Acarbose acts as a suppressor of small intestinal alpha-glucosidase, effectively augmenting the presence of undigested carbohydrates that reach the colon. This increases the production of butyrate, a type of short-chain fatty acid. Acarbose also plays a role in lowering the pH value in the intestinal lumen, by decreasing bacterial deamination and increasing the utilization of ammonia [98].

Keto analogues have been utilized in the treatment of CKD for a significant period of time. These substances have the ability to combine surplus amino acids and facilitate the synthesis of essential amino acids through transamination. This process helps in reducing the accumulation of uremic toxins and can potentially minimize endogen protein degradation, improving kidney function and overall prognosis for the patient [99].

### 3.2. Probiotics for Microbiota Modulation in Pediatric CKD

Probiotics are living bacteria that, when administered, provide health benefits [52,100]. The main probiotic strains typically belong to the genera Bifidobacterium, Lactobacillus, and Streptococcus [52,100]. While there is evidence suggesting the beneficial effects of certain probiotic microorganisms on adult chronic kidney disease (CKD) [101], research regarding their role in pediatric CKD is scarce.

Probiotics exert their effects through various mechanisms, which include altering gut pH, generating antibacterial compounds that combat pathogens, competitively excluding pathogens from binding sites, and binding harmful mutagens and carcinogens [52,99]. These actions contribute to the overall beneficial impact of probiotics on gut health and overall well-being. Although numerous studies discuss the intervention of probiotics in the modulation of the microbiota in adults with CKD, references to children are rare. However, a recent study by Yamaguchi showed that supplementing the diet with butyrate-producing bacteria has a beneficial effect on the evolution of nephrotic syndrome [102]. Tain et al. in their recent review mentions probiotics as microbiota modulators, alongside prebiotics, postbiotics, fecal microbiome transplantation, and bacterial metabolite modulation [52].

### 3.3. Prebiotics for Microbiota Modulation in CKD

Prebiotics are selectively fermented fibers that provide beneficial effects by stimulating the growth and/or activity of intestinal microbes [52,103]. Substances like inulin, fructo-oligosaccharides, resistant starch, and soluble fiber have the potential to decrease uremic toxins and foster the proliferation of good bacteria in individuals with CKD [52]. Furthermore, certain prebiotic-rich diets, such as resveratrol and garlic oil, have demonstrated protective efficacy against CKD in children [103].

### 3.4. Postbiotics for Microbiota Modulation in CKD

The substances derived from bacteria, are known as postbiotics, with various health benefits [104]. They may play a role in maintaining the integrity of the intestinal barrier, reducing inflammation and regulating blood sugar levels. Commonly encountered metabolites include short chain fatty acids (SCFA) such as acetate, propionate, and butyrate [44,52,105]. In animal models, the protective effects of SCFA supplementation as a postbiotic have been investigated in acute kidney injury and chronic kidney disease [52]. However, the utilization of postbiotics in pediatric kidney disease is currently lacking sufficient information.

### 3.5. Fecal Microbiota Transplantation for Microbiota Modulation in CKD

Initiated into clinical practice for the treatment of *Clostridium difficile* infection, transplantation of large feces is garnering increasing interest for the treatment of autoimmune diseases and intestinal dysbiosis [52,106]. Fecal microbiota transplantation (FMT) is an effective approach to restoring the diversity and structure of the microbiota by transferring healthy gut microbiota to recipients. Extensive research has been conducted on FMT for various clinical disorders associated with the microbiome, in both adult [107] and pediatric populations [108]. 

The benefit of facilitating a more robust and long-lasting community of beneficial microorganisms for the body, however, comes with the risk of accidental contact with pathogenic species or the transfer of antibiotic resistance, which must be considered when proposing this therapy [109].

However, there is limited data on the use of FMT in humans with kidney disease.

### 3.6. Colon Dialysis for Microbiota Modulation in CKD

The colon has a crucial role in nitrogen waste elimination, so it can therefore serve as a therapeutic target for the management of CKD. The concept of extra-renal metabolic waste elimination via living membrane barriers is not novel. In Dioscorides’ *Materia Medica* from 40 B.C., the colon was used for the first time as a treatment for kidney disease. Oral absorbents such as oxidized starch have been utilized to remove waste nitrogen from the digestive tract [110]. In the 1970s, Dr. R.D. Rosin experimentally introduced colon dialysis as a simple substitute for hemodialysis and peritoneal dialysis in sheep, proving the usefulness of this method. More recently, in 2014, Iranian physicians utilized colonial dialysis to treat a uremic girl [111]. The colon can therefore serve as a therapeutic target for the management of CKD. On 88 patients, Dai et al. demonstrated in 2019 that colon dialysis is as efficacious as hemodialysis or peritoneal dialysis in stages 4–5 of CKD [112]. Yueming Li et al. carried out a study in 2021 that suggests colon dialysis can keep the kidneys working in stages 3–5 of CKD (before dialysis) by preventing dysbiosis of the intestinal microbiota [113]. This may help to explain the significance of intestinal toxins for the development of CKD, as Zupcic et al. recently demonstrated [114].

## 4. Conclusions

In recent years, the literature has provided evidence for the association between microbial dysbiosis and pediatric kidney disease. Significant findings have mainly focused on the loss of microbiota diversity, alterations in key taxa (such as butyrate-producing bacteria), and changes in gut microbiota-derived metabolites (including SCFA and TMAO). While a substantial amount of evidence has been gathered linking gut microbiota and kidney disease in adult patients, there is a notable lack of such information in the pediatric field. Therefore, large-scale multicenter studies focused on pediatric kidney disease are necessary to establish a definitive connection between the microbial community and kidney disease in the children.

## Data Availability

No new data were created.

## Abbreviations

BC	before Christ
CKD	chronic kidney disease
DNA	deoxyribonucleic acid
DOHaD	Developmental Origins of Health and Disease
ESKD	end-stage kidney disease
FMT	fecal microbiota transplantation
IAA	indole-3-acetic acid
IgA1	Imunglobuline a1
IGM	infant gut microbiota
IS	indoxyl sulfate
LPS	lipopolysaccharides
NF-κB	nuclear factor kappa b
NO	nitric oxide
PCS	p-cresyl sulfate
p-CS	p-cresyl sulfate
pro-oxidants	promote oxidation
RNA	ribonucleic acid
ROS	reactive oxygen species
RTRs	renal transplant recipients
SCFA	short-chain fatty acids
TH17	t helper 17
TLR4	Toll-like receptor 4
TMAO	trimethylamine n-oxide
TNF	tumor necrosis factor
Treg	regulatory t cell
UTI	urinary tract infection
